# Is *Acropora palmata* recovering? A case study in Los Roques National Park, Venezuela

**DOI:** 10.7717/peerj.1539

**Published:** 2016-01-28

**Authors:** Aldo Croquer, Francoise Cavada-Blanco, Ainhoa L. Zubillaga, Esteban A. Agudo-Adriani, Michael Sweet

**Affiliations:** 1 Estudios Ambientales, Universidad Simón Bolívar, Caracas, Venezuela; 2 Biología de Organismos, Universidad Simón Bolivar, Caracas, Venezuela; 3 Environmental Sustainability Research Centre, College of Life and Natural Sciences, University of Derby, Derby, United Kingdom

**Keywords:** Status, Recovery, Threats, Surveys, Los Roques, *Acropora palmata*

## Abstract

Eight years ago (2007), the distribution and status of *Acropora palmata* was quantified throughout Los Roques archipelago in Venezuela. The aim was to produce a baseline study for this species which combined population genetics with demographic data. The results highlighted that *A. palmata* had the potential to recover in at least 6 out of 10 sites surveyed. Recovery potential was assumed to be high at sites with a relatively high abundance of the coral, low disease prevalence, high genetic diversity, and high rates of sexual reproduction. However, as noted, Zubillaga et al. (2008) realized recovery was still strongly dependent on local and regional stressors. In 2014 (this study), the status of *A. palmata* was re-evaluated at Los Roques. We increased the number of sites from 10 in the original baseline study to 106. This allowed us to assess the population status throughout the entirety of the MPA. Furthermore, we also identified local threats that may have hindered population recovery. Here, we show that *A. palmata* now has a relatively restricted distribution throughout the park, only occurring in 15% of the sites surveyed. Large stands of old dead colonies were common throughout the archipelago; a result which demonstrates that this species has lost almost 50% of its original distribution over the past decades. The majority of corals recorded were large adults (∼2 m height), suggesting that these older colonies might be less susceptible or more resilient to local and global threats. However, 45% of these surviving colonies showed evidence of partial mortality and degradation of living tissues. Interestingly, the greatest increase in partial mortality occurred at sites with the lowest levels of protection (${X}_{o}^{2}=5.4> {X}_{c}^{2}=4.5$; *df* = 4, *p* < 0.05). This may suggest there is a positive role of small scale marine management in assisting reef recovery. We also recorded a significant reduction (${X}_{\mathrm{exp}}^{2}=126.8> {X}_{\mathrm{cri}}^{2}=15.5$; *df* = 8; *p* < 0.05) in the density of *A. palmata* in sites that had previously been categorized as having a high potential for recovery. One explanation for this continued decline may be due to the fact that over the past 10 years, two massive bleaching events have occurred throughout the Caribbean with records showing that Los Roques has experienced unprecedented declines in overall coral cover. We therefore conclude that although local protection could promote recovery, the impacts from global threats such as ocean warming may hamper the recovery of this threatened species.

## Introduction

The western Atlantic is the second largest coral reef bioprovince on earth (Veron, 1995). This region extends from the eastern coast of Brazil up to Bermuda with the largest and most diverse reefs being found in the Caribbean basin (Birkerland, 1997). During the past five decades, Caribbean coral reefs have been exposed to a great deal of impacts which have challenged their resilience on local and regional scales (Gardner et al., 2003; Gardner et al., 2005; Bellwood et al., 2004). Indeed, in all but only a few exceptions, Caribbean coral reefs have undergone a rapid decline (Jackson et al., 2014). Such accelerated deterioration of reef ecosystems have been attributed to overfishing and the increasing input of nutrients combined with bleaching and disease events that have produced massive die-offs of keystone reefs organisms (Hughes et al., 2003; Pandolfi et al., 2003; Bellwood et al., 2006; Hughes et al., 2010; Jackson et al., 2014). The combination of these local and global threats have significantly reduced populations of major reef-building coral species such as *Acropora palmata* to critical levels, not only threatening the species but also significantly jeopardizing any potential of recovery on already degraded reef systems (Jackson, 2001; Jackson et al., 2001; Gardner et al., 2003; Hughes et al., 2003; Halpern et al., 2007; Knowlton & Jackson, 2008).

The elkhorn coral *Acropora palmata* is a hermaphroditic broadcast spawner that grows 5–10 cm year^−1^ (Gladfelter, Monahan & Gladfelter, 1978) forming complex and heterogeneous reef frameworks in shallow waters (Adey & Burke, 1976; Bak & Criens, 1981; Highsmith, 1982; Szmant, 1986). Since the Pleistocene, *A. palmata* was a widespread and conspicuous coral reef builder throughout most of the Caribbean (Pandolfi & Jackson, 2006; Pandolfi, 2002; Aronson & Precht, 2001). A regional collapse of *A. palmata* populations due to white band disease greatly reduced the abundance of this species throughout its entire distribution range (Gladfelter, 1982; Aronson & Precht, 2001). As a result, in 2006, this species, along with *Acropora cervicornis* were the first two species of corals to be listed under the United States Endangered Species Act as ‘threatened’. In 2008, the species was categorized in the IUCN Red List of threatened species as critically endangered (CR; Aronson et al., 2008) and, almost four decades after the major mortality event, it remains unclear whether populations of *A. palmata* are recovering or continuing to decline. While several studies have shown evidence of moderate recovery in certain locations (Grober-Dunsmore, Bonito & Frazer, 2006; Mayor, Rogers & Hillis-Starr, 2006; Edmunds, 2014; Muller, Rogers & Van Woesik, 2014), an equal number of contrasting studies have shown little or no recovery with low genetic diversity (Japaud et al., 2015) and an estimated decline in abundance across the wider Caribbean reaching up to 97% (Knowlton, Lang & Keller, 1990; Porter et al., 2001; Bruckner, 2002; Rodríguez-Martínez et al., 2014; National Marine Fisheries Service, 2015).

Recently, Marine Protected Areas (MPAs) have been suggested as a means to mitigate or even reverse the decline of marine ecosystems and corals worldwide (Bellwood et al., 2004). However, their success has been shown to vary considerably (Lester et al., 2009; Crabbe, 2015). For example, in Kenya (McClanahan & Muthiga, 1988; McClanahan & Mutere, 1994; McClanahan, 1997) and in the Great Barrier Reef (Williamson, Russ & Ayling, 2004), MPAs have successfully enhanced the abundance or live cover of hard corals. In contrast, coral cover within ‘no-take’ zones on Glovers Reef, in Belize has reported to be lower than in adjacent unprotected reef systems (McClanahan, Muthiga & Mangi, 2001). MPAs have also appeared to fail in mitigating declines in coral cover on reefs in Papua New Guinea (Jones et al., 2004) and the Little Cayman Island in the Caribbean (Coelho & Manfrino, 2007). Thus, the role of MPAs in preventing coral decline remains unclear.

For example, Los Roques is arguably one of the most well-preserved and pristine sites within the Caribbean (Jackson et al., 2014). However, there is limited baseline information regarding the status of coral populations such as *Acropora palmata* (Zubillaga et al., 2008; Porto-Hannes et al., 2015). The initial observations were first conducted in 2005 at Cayo de Agua, where healthy populations were described (Zubillaga, Bastidas & Cróquer, 2005). In 2007, ten further sites were surveyed with the purpose of collecting demographic (i.e., abundance, size structure, partial mortality and prevalence of diseases) and genetic (i.e., allele diversity and patterns of connectivity) data (Zubillaga et al., 2008). This latter study highlighted that *A. palmata* had high potential of recovering in, at least, six sites of the archipelago. This was concluded as the abundance of this species was above the Caribbean standards (i.e., lack or low densities); whereas the prevalence of white band disease (WBD) and partial mortality was low (Zubillaga et al., 2008). The authors also found high allelic diversity, moderate to high levels of connectivity; and more importantly, low proportion of clone mates within these populations. Nevertheless, according to Zubillaga et al. (2008), the combined negative effects of local and global threats might hinder and/or prevent this species from regaining its former status in Los Roques. Consequently, the urgent need of an appropriate management of Los Roques was recognized, given the rapid increase in tourism and other human activities inside this MPA (Zubillaga et al., 2008). However, these former conclusions, about the status of *A. palmata* in Los Roques, came from a limited number of sites and likely did not truly portray the status of the species at the scale of the entire MPA. This limits the design and application of any effective conservation strategy (Aswani et al., 2015). Herein, we determined the current distribution, abundance, and health status of *A. palmata* populations in Los Roques at the scale of the MPA. We also compared our results to previous assessments of the species and to levels of protection.

## Material and Methods

### Study area

**Figure 1:**
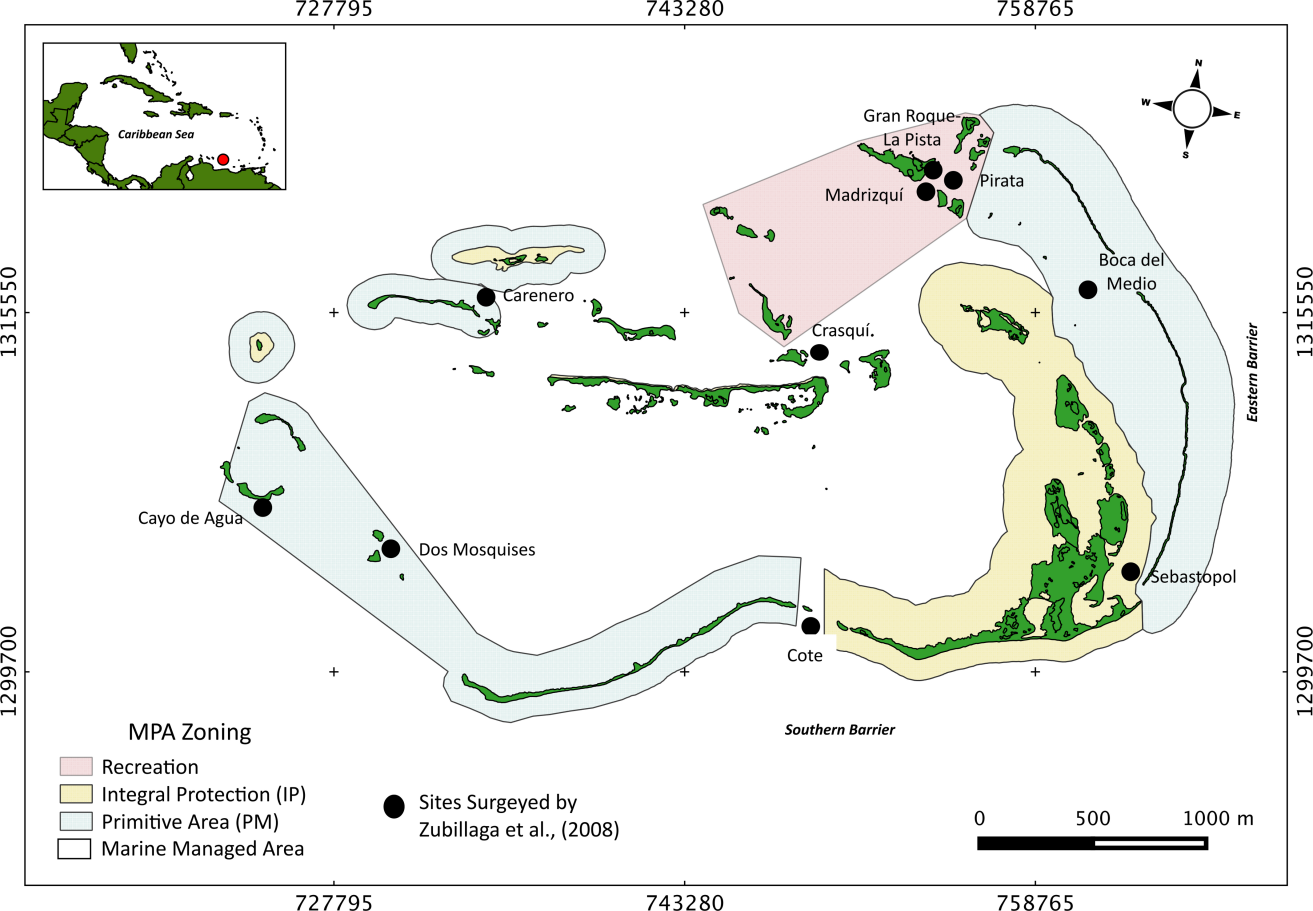
Study site. Map of Los Roques National Park (ALRNP) and coastal-marine zoning of the MPA.

The Archipelago Los Roques National Park (ALRNP) is an oceanic archipelago located 160 km north off the Venezuelan coast (REGVEN/UTM 19N 721011-7671071324721-1297746; Fig. 1). The reef system encompasses more than 50 coral cays with fringing reefs, patch reefs, over 200 sand banks, and extensive mangrove forests and seagrass beds (Weil, 2003). ALRNP was the first MPA in Latin America, decreed as a National Park in 1972. In 1991, the zoning and use regulations were established prioritizing the protection of marine turtles and migratory birds nesting sites. The MPA zoning encompasses nine different use-zones, from which four include coastal-marine habitats, making ALRNP a multi-use MPA. These zones range from high protection (Integral Protection Zone, (IP) and Primitive Zone, (PM)), wherein only scientific research or managed non-extractive activities are allowed, to medium (Marine Managed Area, (MMA)) and low protection (Recreational (R)) levels. In these latter two zones, recreational activities and artisanal fisheries are permitted. According to this zoning, human activities are mostly concentrated within the northeast main island, (Gran Roque), and nearby cays (Fig. 1).

### Surveys of *Acropora palmata*

To determine the current distribution and abundance of *Acropora palmata*, visual censuses were conducted between April and November 2014, encompassing 106 sites across the archipelago. These sites were selected to cover the vast majority of locations and habitats within the MPA. Several criteria were used during the selection, including: (1) personal expertise and knowledge of the MPA, (2) anecdotal information collected from the local populace and (3) observation of potential habitats from raster satellite images. With these criteria, the surveys not only included areas dominated by *A. palmata*, but also covered a suite of different habitats including windward (exposed) and leeward (protected) cays, fringing and barrier reefs, reef patches and mixed seagrasses and sand habitats within the lagoon. From this data, a map of distribution for *A. palmata* was able to be produced at the scale of the entire MPA.

At each site, four observers conducted the visual surveys by doing free dives along shallow to intermediate habitats (1–12 m depth). Twenty (20) m wide belt-transects were surveyed ranging from 400 to 1,000 m in length. The length and the number of the belt-transects varied according to the extent of the same type of habitat at each location and during each dive. At each belt-transect the start and end points were geo-referenced with a Garmin 60S GPS. Within each belt-transect, every colony was counted and basic information associated with the colonies adjacent habitat was recorded (e.g., depth, slope and level of wave exposure). For each colony, a visual inspection was also conducted to determine the presence/absence of partial mortality and disease signs. Every sign of tissue discontinuity (recent or old), disease and/or health problems (i.e., white band, white spot or patchy necrosis) was annotated using ID cards (Weil & Hooten, 2008). Dead stands of *A. palmata* as well as recent and poorly eroded deposits were also recorded as a proxy of former distribution range of this species within the MPA.

Habitat characterization was assessed qualitatively, on the basis of direct observations of predominant substrates (e.g., sandy bottoms, consolidated reefs, pavement, etc.), dominant benthic organisms (e.g., hard corals, gorgonians and sponge), and the presence/absence of massive (e.g., *Colpophyllia* spp, *Diploria* spp, *Pseudodiploria strigosa*, *Orbicella* and *Montastrea* sp), branching (e.g., *Madracis* spp, *Acropora* spp, *Porites* spp), foliaceous (e.g., *Agaricia* spp) and encrusting (e.g., *Agaricia agaricites*) hard coral as well as gorgonian species (e.g., *Gorgonia* spp, *Pseudopterogorgia* spp, *Pseudoplexaura* spp, *Plexaura* spp, *Eunicea* spp). This rapid characterization was performed using similar procedures for plotless belt-transects outlined by English, Wilkinson & Baker (1997) modified with random free dive instead of scuba diving. While this method has relatively low precision at small spatial scales, it is suitable for rough descriptions of the coral benthic communities for broader spatial scales as in this study (Hill & Wilkinson, 2004).

Habitat health status was classified into four arbitrary categories according to the relative predominance of live coral cover, macro-algae, bare substratum and sedimentation. These included: (1) ‘Excellent’ (i.e., the corals clearly dominated the benthos, with no evidence of sedimentation and/or coral mortality), (2) ‘Good’ (corals still dominated, yet sedimentation was evident but dead corals were rare), (3) ‘Regular’ (corals and macro-algae have similar abundance and sedimentation was present, bared substratum was common) and (4) ‘Degraded’ (macro-algae clearly dominated the benthos with sediment often smothering the corals and denuded substratum was abundant).

All permits necessary to conduct this work were processed and accepted by the Governmental Venezuelan authorities (i.e., Ministerio del Poder Popular para el Ambiente-Oficina de Diversidad Biológica). Oficio No. 0323 and Territorio Insular Francisco de Miranda. Autorización Provisional No. 006.

### Local threats distribution

In our analysis we included four main categories regarding local threats; land-based pollution (LBP): for example, pollution from Gran Roque Island where the largest human population in Los Roques is settled and where the majority of solid and hydrocarbon wastes are produced; touristic beach activities (TBA), diving (D) and spiny lobster fishing grounds (F). Information about such threats, was obtained through interview questionnaires and semi-structured interviews administered to different stakeholders (Schnell & Kreuter, 2003; F Cavada-Blanco, 2014, unpublished data). All interviewers were previously trained before conducting the interviews.

The location of lobster fishing grounds was determined by conducting interviews with fisherman in which a map with a 20 m^2^ cell grid was shown to them during the spiny lobster season. Impacts from fishing were not included in the analysis for fishermen do not use nets and seldom fish inside the archipelago and/or *Acropora palmata* grounds. The specific locations of touristic beach activities (TBA) were determined based on the most visited sites reported by lodge staff. This data was then further corroborated by touristic transportation cooperatives. The precise location of dive sites was collected from dive instructors and dive masters. Respondents were selected on the basis of willingness to be interviewed. The surveyed sampling size varied depending on the size of each stakeholder group. Thus, we surveyed 100% of dive operators (*N* = 3); 58.3% of lodges (*N* = 35), 100% of touristic transportation cooperatives (*N* = 2) and 35% of licensed fishermen (*N* = 200).

### Spatial analysis and species representativeness

To assess representativeness of *Acropora palmata* according to the protection level of the MPA zoning (i.e., the set of sites within the MPA including the species of interest), we performed a GAP analysis (Jennings, 2000) using the species distribution and MPA zoning as layers. A vector layer of the total area of dead stands was also built using this as a proxy to evaluate the area whereby habitat loss has occurred within the archipelago. To visualize the spatial distribution of local anthropogenic threats and to determine the distance between the occurrence of *A. palmata* and these specific threats; a distance matrix was calculated using the centroids of areas occupied by *A. palmata* as the input layer and each threat as target layers. Layers were built and visualized using QGIS 2.4 Chugiak and spatial analysis was performed using the R package “sp” (Pebesma et al., 2012).

### Statistical analysis

In order to test if the density of *Acropora palmata* recorded in this survey (2014) to that occurring in 2007 had changed; a Chi–Square test was conducted, considering coinciding sampled sites from both survey periods. For this, a two-column (year) × 9 row (sites) contingency table was utilized (Sokal & Rohlf, 1995). Colony counts for each site were standardized by area covered for both years. Similarly, another contingency table was constructed in order to test whether the probability of occurrence of healthy colonies and the ones showing partial mortality were independent of the zoning. In this case the data was standardized by the number of sites surveyed within each zone. The same procedure was used to test whether the distribution of ‘Degraded’, ‘Regular’, ‘Good’ and ‘Excellent’ habitats was independent of the zoning. A non-metric multidimensional scaling and analysis of similarities (ANOSIM, Clarke & Green, 1988) based on Euclidean distance was performed to test whether the average distance of local threats to the occurrence of *A. palmata* populations differed across management zones.

## Results

### Abundance and distribution of *Acropora palmata* across ALRNP

**Figure 2:**
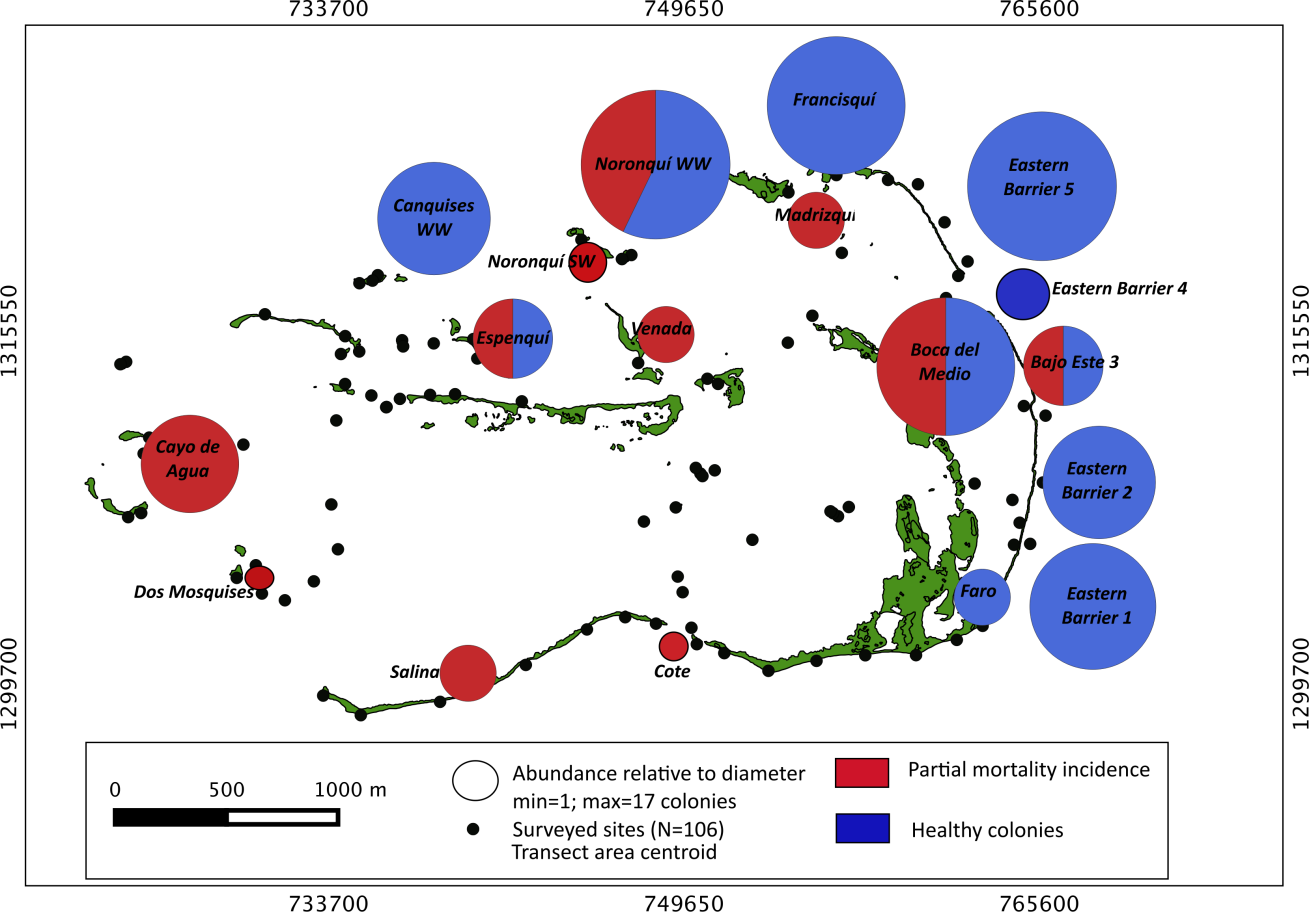
*Acropora palmata*. Abundance and health status. Abundance and health status of *Acropora palmata* across sites within the ALRNP.

**Figure 3:**
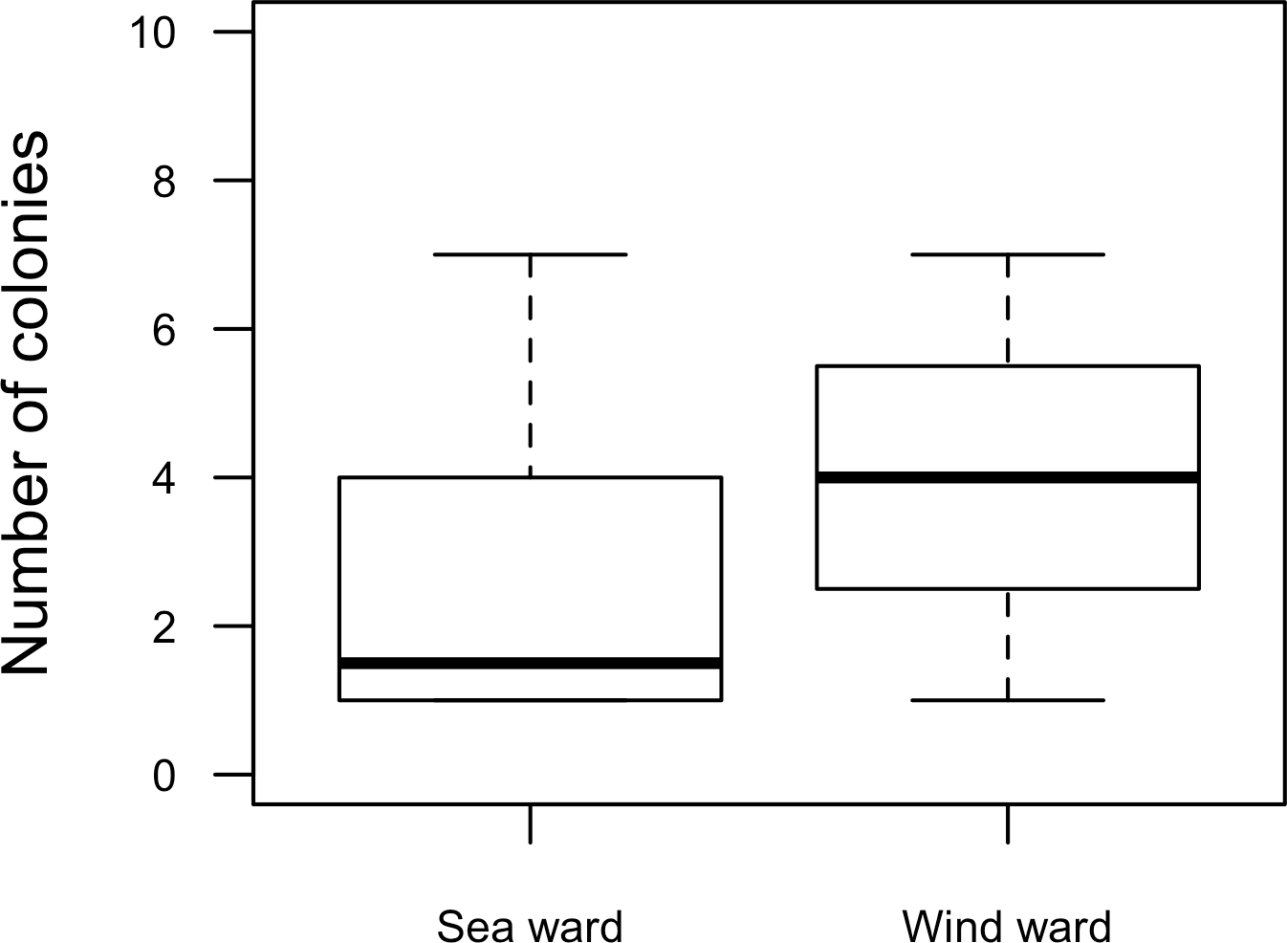
*Acropora palmata*. Habitat. Abundance and health status of *Acropora palmata* across sites within the ALRNP.

Our surveys covered a total area of 6.72 km^2^. In total, only sixty-seven live *Acropora palmata* colonies were observed, and only on 15% of the surveyed sites (Fig. 2). This represented an area of occurrence of 134,800 m^2^ or 6% of the total area of the MPA. The frequency of occurrence was extremely low (∼10 colonies/km^2^), with density values ranging from 0.001 to 0.1 live colonies per 100 m^2^. The prevalence of recent partial mortality was conspicuous, ranging from 33 to 100% of the colonies across sites. The exception to this pattern was only observed in the exposed reefs along the eastern barrier, where colonies showed no signs of partial mortality (Fig. 2). On average, the majority of the colonies were located on seaward reefs (61.2%), although the number of colonies varied greatly in relation to wave exposure (2.5 and 0.9 times the average, respectively, Fig. 3). The results indicated that *A. palmata* has a very restricted distribution range in Los Roques with healthy populations further limited to only a few sites.

**Figure 4:**
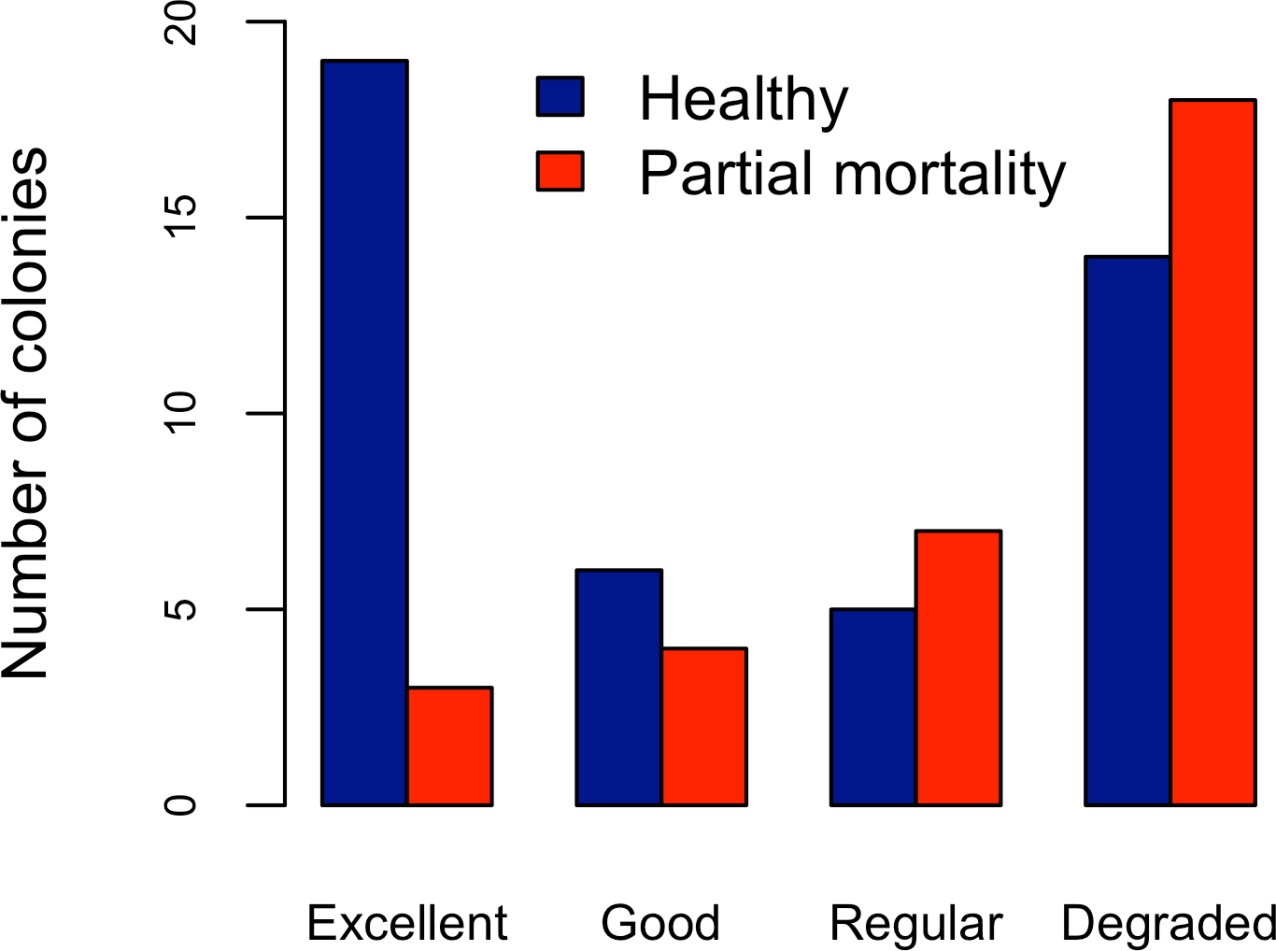
*Acropora palmata*. Health Status and habitat status. Abundance of *Acropora palmata* colonies according to health status of the habitat.

**Figure 5:**
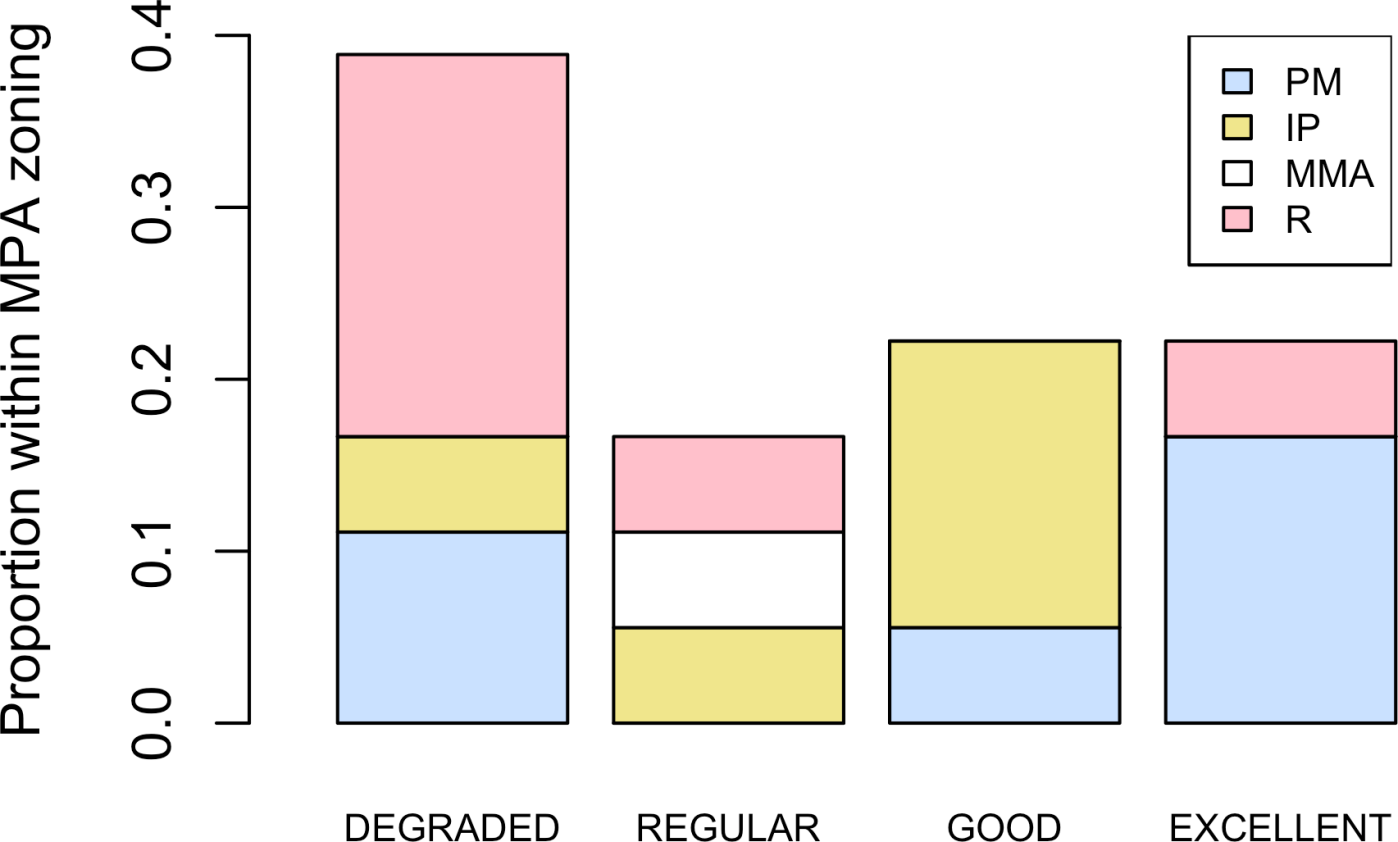
Zoning and habitat status. Distribution of habitats with different health status across protection zones.

Five different types of habitats were documented (Table 1). The most frequent and characteristic habitat of *A. palmata* in Los Roques was composed of pavement substrate, dominated by *Pseudoplexaura* spp. and *Plexaura* spp. with scattered colonies of *Diploria labyrinthiformis*, *Pseudodiploria clivosa*, *Colpophyllia natans* and *Porites astreoides* (Table 1). Over 50% of the colonies occurred in habitats categorized either as being in ‘Excellent’ (26.7%) or ‘Degraded’ (33%) conditions (Fig. 4). These results indicate that in many of the sites surveyed, the habitat of *A. palmata* is degraded. However, in other sites, reefs still remain healthy. We found no significant association between the quality of the habitat and the level of protection (${X}_{o}^{2}=11.6> {X}_{c}^{2}=16.9$; *df* = 9, *p* < 0.05; Fig. 5).

**Table 1:** *Acropora palmata* habitat types in Los Roques National Park.

Main substrate	Dominant species/group	Description
Sand	*Acropora cervicornis*	Sand flats with dense patches of *Acropora cervicornis*, and *A. cervicornis* rubble. Presence of scattered massive coral species such as *Diploria labyrinthiformis, clivosa* and *strigosa*, *Colpophyllia natans*, *Orbicella annularis* and *Porites astreoides*. Few gorgonians such as *Pseudopterogorgia* spp and *Gorgonia ventalina*. Calcareous algae such as *Halimeda* sp and *Turbinaria* sp are rare.
	Scattered massive corals	Sand flats or smooth slopes with scattered large (more than 1 m height) colonies of *Diploria labyrinthiformis, clivosa* and *strigosa*, *Colpophyllia natans*, *Siderastrea siderea*, *Orbicella annularis, Porites porites* and *Porites astreoides*. Calcareous algae like *Halimeda* sp and *Turbinaria* sp are also present.
	Soft corals	Sand flats with dense patches of *Pseudopterogorgia* and *Plexaura flexuosa* with scattered colonies of *Diploria strigosa* and *Orbicella annularis* mixed with octocoral patches.
Consolidated reefs	*Orbicella annularis*	Flat or smooth slopes of large and consolidated (<2 m height) *Orbicella annularis* colonies (no distinction can be made between ramets and genets) with scattered colonies of *Acropora cervicornis*, *Diploria labyrinthiformis, clivosa* and *strigosa*, *Colpophyllia natans*, *O. faveolata* and *Porites astreoides*. Fewer gorgonians such as *Pseudopterogorgia* spp and *Gorgonia ventalina* and abundant incrusting and tube-like sponges. Abundant calcareous algae.
Pavement		With or without rubble. Presence of scattered coral species like *Diploria labyrinthiformis, clivosa* and *strigosa*, *Colpophyllia natans*, *Orbicella annularis* and *Porites astreoides, A. cervicornis* and *palmata* growing as incrusting morphotypes. Few soft corals like *Pseudopterogorgia* spp and *Gorgonia ventilata*. Presence of calcareous algae like *Halimeda* sp and *Turbinaria* sp.

**Figure 6:**
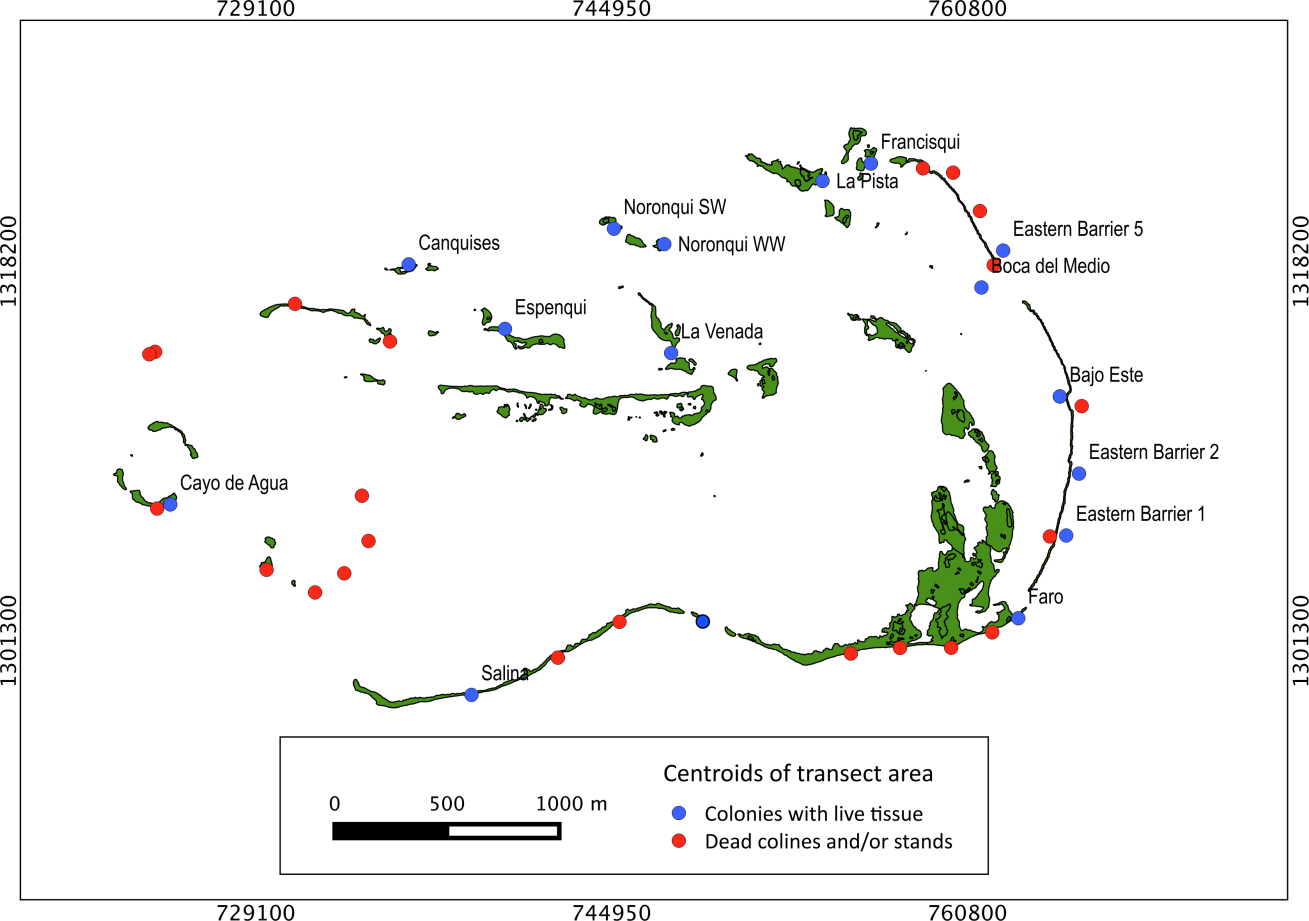
Historical distribution of *Acropora palmata*. Occurrence of live and dead stands of *Acropora palmata* across ALRNP.

Extensive dead stands of *A. palmata* were counted on 23% of the surveyed sites, which represents a 51.3% loss of the historic distribution on the archipelago (Fig. 6). Indeed, a significant reduction (${X}_{o}^{2}=126.8> {X}_{\mathrm{cri}}^{2}=15.5$; *df* = 8; *p* < 0.05) was also recorded in the density of *A. palmata* found in 2008 and those found during this survey in 2014 (Table 2). Combined these results show, that at the scale of the MPA, *A. palmata* distribution has shrunk over the past decade (Fig. 6), with the population declining over the past seven years (Table 2).

**Table 2:** *X*^2^ test comparing the number of colonies of *Acropora palmata* in 100 m^2^ at nine sites in 2007 (data from Zubillaga et al., 2008) and 2014.

${X}_{\mathrm{ob}}^{2}=126.8$, ${X}_{c}^{2}=15.5$, *df* = 8, *p* < 0.05				
	2007		2014	
Sites	Total	Density (col/100 m^2^)	Total	Density (col/100 m^2^)
Cayo de Agua	257	3.213	3	0.007
Herradura de Dos Mosquises	137	1.713	10	0.1
Gran Roque	73	0.913	1	0.003
Crasquí	64	0.800	1	0.001
Cayo Pirata	55	0.688	0	0.000
Sebastopol	10	0.125	1	0.001
Madrisky	10	0.125	0	0.000
Maceta de Cote	4	0.050	2	0.037
Boca del Medio	3	0.038	6	0.021

### *Acropora palmata* representativeness and local threats distribution

**Figure 7:**
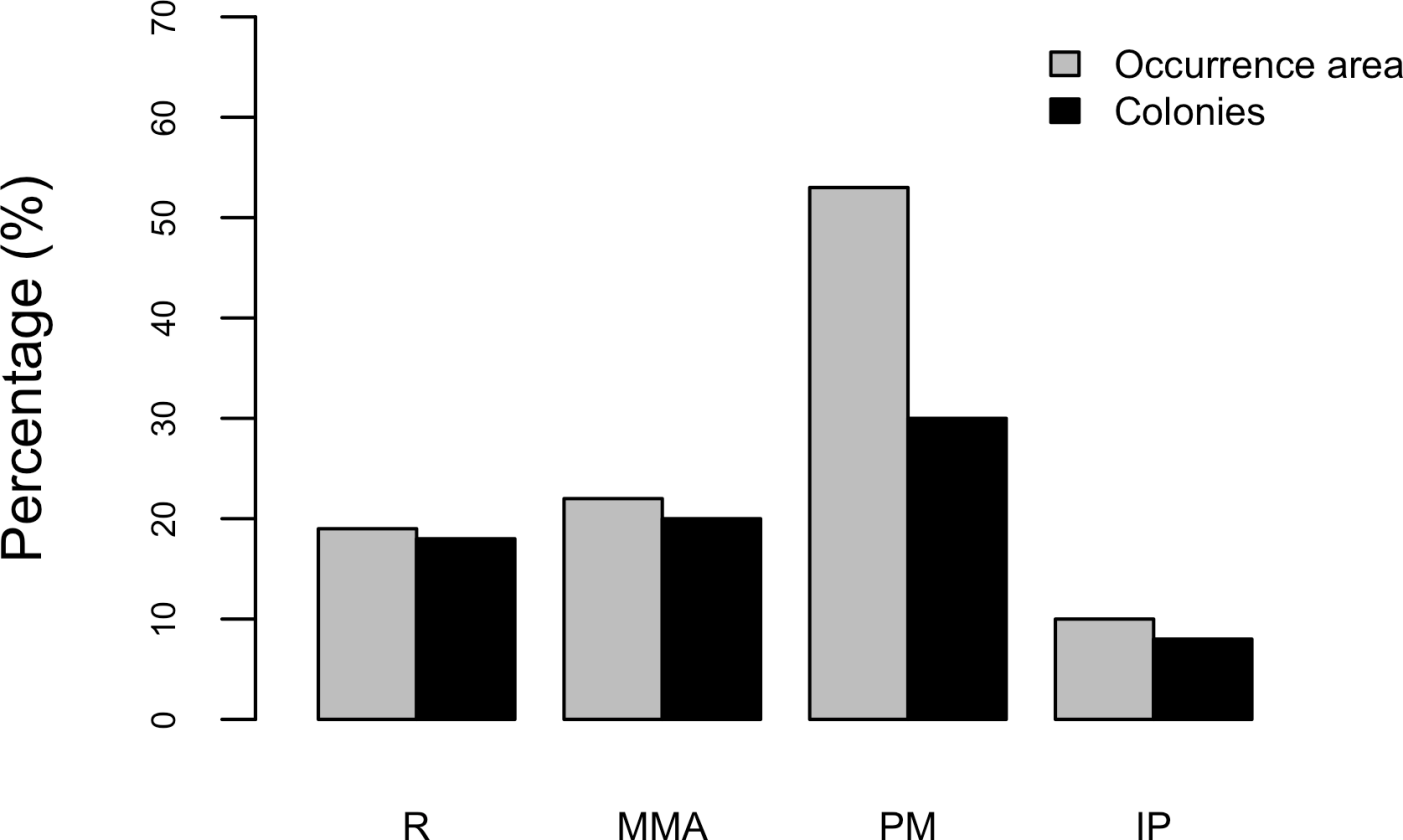
*Acropora palmata* occurrence and zoning. Occurrence of *Acropora palmata* across different protection zones.

**Figure 8:**
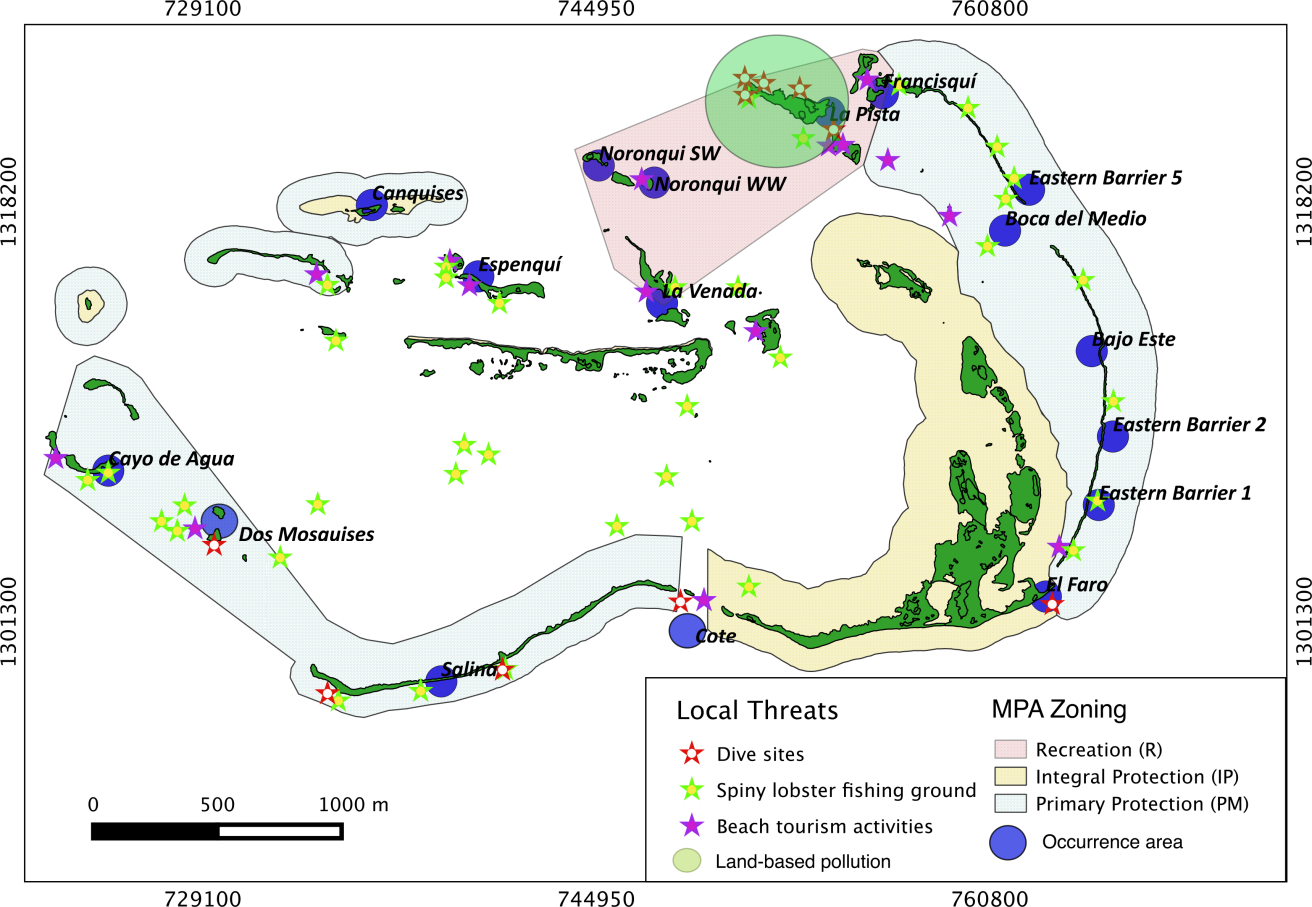
*Acropora palmata* and local threats. Spatial distribution of *Acropora palmata* against local threats within ALRNP.

**Figure 9:**
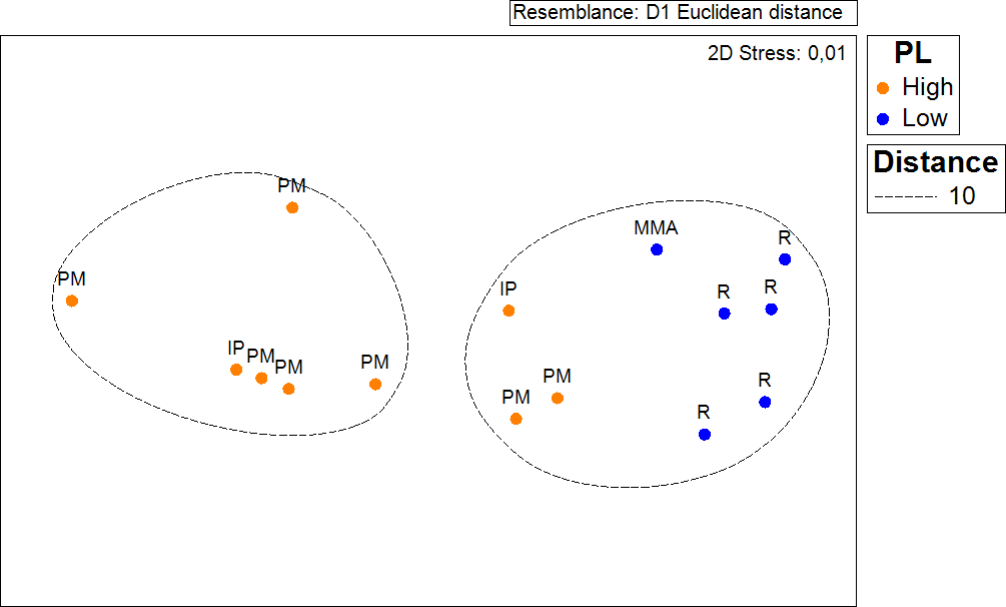
ANOSIM and nMDS. Non Metric Multidimensional Scaling showing the ordination of *Acropora palmata* sites observed across using zones within the ALRNP and their distance from 4 local threats: (1) tourism, (2) diving, (3) lobster fishing grounds and (4) land-based pollution. Sites with low protection are significantly closer to these threats compared to sites with highly-protected ANOSIM (*R* = 0.74, *p* = 0.02).

Results show that 61% of the total occurrence area of *Acropora palmata* is located in the highest protected zones, whereas 29.7% were observed inside the least protected areas such as the recreational and marine managed zones (Fig. 7). Furthermore, the frequency of partial mortality incidence was significantly (${X}_{o}^{2}=5.4> {X}_{c}^{2}=4.5$; *df* = 4, *p* < 0.05) greater within the least protected areas. Within the recreational zone, *A. palmata* was commonly found at Francisquí and La Pista in Gran Roque. Both these sites are less than 1 km apart from one of the most visited diving sites (Madrizquí) and also from the main source of land-based pollution. *A. palmata* was also frequent at Noronquí, La Venada and Espenquí, all of which are less than 5 km apart from heavily populated and touristic sites. However, most of the touristic, diving and lobster fishing activities were also carried out inside the integral protection and primitive zones (Fig. 8). Inside these protected zones, 85% of sites with *A. palmata* were less than 5 km from at least one of the threats considered in this study; the only exception being that of land-based pollution (Table 3). In fact, the nMDS showed that on average, populations of *A. palmata* located at areas with low protection (e.g., recreational sites) were significantly closer to local threats (ANOSIM *r* = 0.74, *p* = 0.02) compared to highly-protected areas (Fig. 9).

**Table 3:** Average distance as centroids between *Acropora palmata* occurrence sites and identified local threats.

Occurrence sites	Threats			
	Fishing grounds	LBP	Diving sites	Touristic activities
Bajo Este	21.08	17.52	20.83	19.84
Boca del Medio	19.01	12.79	18.26	16.80
Canquises	17.23	15.81	19.10	16.08
Cayo de Agua	21.75	27.31	25.23	23.73
Espenqui	14.33	13.01	16.16	13.13
Faro	22.09	22.37	21.46	21.64
Francisqui	18.92	8.20	15.87	15.10
La Pista	17.77	7.39	14.18	13.81
La Venada	13.42	9.54	13.77	11.45
Noronquí SW	15.74	10.25	14.86	12.88
Noronquí WW	15.29	9.08	13.93	12.19
Salina	17.56	23.70	18.90	20.27
Eastern barrier 1	22.17	21.14	21.92	21.50
Eastern barrier 2	22.03	19.91	21.83	21.17
Eastern barrier 5	20.11	13.30	19.19	17.86

**Notes.**

LBP, Land-based Pollution.

## Discussion

Here we present results from a survey of *Acropora palmata* conducted at ALRNP, covering an area of more than 6.7 km^2^ over 106 sites. This study complements previous reports and gives a more complete understanding of the status of *A. palmata* throughout ALRNP by evaluating the species representativeness according to the MPA zoning and identified local threats. Although comparisons with previous assessments of the species in ALRNP allows determination of a general trend in abundance, this study represents the first baseline assessment of *A. palmata* at the MPA scale. Similar to previous reports (Zubillaga et al., 2008), in this broader scale assessment, we found large stands of old-dead *A. palmata* cemented and covered by calcareous coralline algae across 23% of sites surveyed. This indicates a long-term shrinkage of *A. palmata* distribution within ALRNP over the past few decades. Furthermore, the analysis of *A. palmata* occurrence at the scale of the whole MPA indicated that the species currently has a very limited distribution within ALRNP (only 6% of the total MPA area). We found a significant decrease in the population number of this species in sites where larger population sizes were reported in 2008. Furthermore, about 30% of the occurrence area of this species overlapped with the presence of local threats such as land-based pollution, tourism and lobster fishing within the MPA. This result suggests that local threats might not be limiting the distribution of this species within Los Roques.

Our results indicate that ALRNP might no longer be one of the few exceptions to the regional declines this species has exhibited throughout its range (Zubillaga et al., 2008). Densities, ranging from 0.05 to 0.001 colonies/100 m^2^ were recorded, displaying variable but frequent partial mortality. Such densities are two–three orders of magnitude less than those reported in 2008 (i.e., 0.4–32 colonies × 100 m^2^, Zubillaga et al., 2008). Similarly, declines of upwards of 97% have occurred within the region, such as the Florida Keys, Jamaica, Dry Tortugas, Belize, St Croix and Puerto Rico (Acropora Biological Review Team, 2005; Weil, 2003). The densities reported in our study do reflect those reported across the majority of the Caribbean. For example, between 80 and 98% of *A. palmata* colonies have been recorded as being lost since 1980. In this region in particular, the density of *A. palmata* has remained below 10 colonies per 100 m^2^ since its initial decline (Bruckner, 2002; Acropora Biological Review Team, 2005; Rodríguez-Martínez et al., 2014). Densities above these have only been reported for relatively few sites. These include, Florida: 80–100 colonies per 100 m^2^ (Bruckner, 2002), Mexico: 76 colonies per 100 m^2^, Colombia: 60 colonies per 100 m^2^ (Jordán-Dahlgren, 1992) and previously, ALRNP: 32 colonies per 100 m^2^ (Zubillaga et al., 2008). In specific sites such as Cayo de Agua, Dos Mosquises, Madrizquí and Carenero, densities of *A. palmata* declined by 1–2 orders of magnitude when compared with 2008 reports (Zubillaga et al., 2008).

While we have not directly addressed in this study the reason or reasons for recent *A. palmata* mortality, the only event recorded in recent years (which has had proven impacts on coral communities in ALRNP) was the 2010 bleaching event (Bastidas et al., 2012). During the 2010 bleaching event, SST in Los Roques stayed above 30°C for a few weeks (Bastidas et al., 2012). Such extensive mortality on *A. palmata* and other scleractinian populations after massive bleaching and epizootic events has been reported in other areas such as the US Virgin Islands (Muller et al., 2008; Brandt & McManus, 2009; Miller et al., 2009) and the Florida Keys (Williams & Miller, 2012). It is therefore possible that ocean warming and subsequent bleaching and disease (Rosenberg & Ben-Haim, 2002; Miller et al., 2009) caused the observed reduction in abundance and health condition of *A. palmata* throughout the ALRNP.

A recent study by Randall & Van Woesik (2015) quantified the effects of ocean warming on the prevalence of a particularly devastating disease affecting both *A. palmata* and *A. cervicornis*. They found that decadal warming in sea surface temperature (SST), increases in thermal minima, and the breach of thermal maxima have all played significant roles in WBD outbreaks since 1997. As temperatures continue to increase, vulnerability thresholds (28.5°C for *A. palmata*) will be breached more frequently, resulting in an increased bleaching and disease (Randall & Van Woesik, 2015).

Interestingly, wave-exposed sites along the eastern barrier in the PM zone still held healthy colonies, with the highest observed densities of *A. palmata*. While this species was observed in sandy bottoms and sheltered-shallow sites as reported before in ALRNP (Zubillaga et al., 2008) and across the whole Caribbean (Acropora Biological Review Team, 2005), the majority of the colonies throughout the archipelago were more commonly associated with consolidated substrates such as pavements which in turn are located in wave-exposed habitats. Survivorship of *A. palmata* colonies has been previously shown to be higher in these same habitats compared to those associated with unconsolidated bottoms (Lirman, 2000). This pattern might suggest two non-mutually exclusive alternatives: (1) that in habitats with greater wave exposure, thermal stress during the bleaching event was less severe and shorter than in sheltered sites and/or (2) that regardless of the severity of the thermal stress, wave-exposed areas recovered faster. Indeed, it has been well documented that reef scale oceanographic and geomorphologic characteristics affect both biological and physical variables which in turn influences the severity of bleaching events (Jokiel & Brown, 2004). Furthermore, these same characteristics have also been linked to recovery rates of bleached coral populations (Golbuu et al., 2007).

Regarding the effectiveness of the MPA in mitigating temperature-driven degradation of coral reef and reef-building coral populations, in this instance there appears to be little effect, especially during acute bleaching events (Page et al., 2009; Monzón, Moyer-Horner & Palamar (2011); Selig, Casey & Bruno, 2012). However, the survey methodology implemented in this study does not permit us to discern between the true effects that the level of protection and aspects of habitat heterogeneity such as wave-exposure has on the spatial pattern of abundance, partial mortality incidence and degraded habitats (Miller & Russ, 2014). However, this study does show that the incidence of colonies showing partial mortality is lower in areas with medium to strong protection. This result suggests that MPA zoning may indeed help to protect this species, at least in some manner. MPAs are generally regarded as the ‘gold standard’ for reef preservation and assisting recovery after any such disturbance (Heller & Zavaleta, 2009; McLeod et al., 2008; Graham et al., 2014). Nevertheless, empirical evidence on the success of MPAs for coral recovery is at current non-conclusive (McClanahan, 2008; Graham, Nash & Kool, 2011; Halpern, White & Aswani, 2012; McClanahan et al., 2012). It seems likely that increasing protection efforts in such locations as ALRNP may not necessarily result in *A. palmata* escaping any further massive mortality events during instances of mass-bleaching for example. Yet, MPAs such as Los Roques may still offer some respite against intermittent global threats by controlling the negative effects of more localised threats.

This is still important for regional populations, as local threats, such as those identified in this study, are recognized as major threats to the remaining populations in certain areas such as the Florida Keys and the US Virgin Islands (for example Patterson et al., 2002; Bythell, Pantos & Richardson, 2004; Sutherland & Ritchie, 2004; Grober-Dunsmore, Bonito & Frazer, 2006; Wirt et al., 2015). Some of these threats are still infrequent at Los Roques (e.g., diseases, snails, storms and hurricanes); however, human pressure, such as land-based pollution, algal blooms and parrotfish exploitation seem to have increased during recent years and might be becoming an increasingly serious problem (F Cavada-Blanco, unpublished data). In Los Roques, the distribution of *A. palmata* overlapped with common local threats such as diving (Hawkins & Roberts, 1992; Hawkins et al., 2005; Barker & Roberts, 2004), tourism (Hawkins & Roberts, 2004) and land-based pollution, which are all known to have serious deleterious effects on many coral species on a global scale (Fabricius, 2005). According to Aronson et al. (2008) the loss of habitat at the recruitment stage due to algal overgrowth and sedimentation; predation by snails; mortality by endolithic sponges; ship groundings, anchor damage, trampling, and marine debris have all been responsible for local demise of *A. palmata*.

To conclude, our results indicate that *Acropora palmata* has a restricted distribution across the ALRNP with the majority of the original distribution already being lost. Healthy populations of *A. palmata* can still be found along the eastern barrier and at a limited number of sites across the archipelago, however, significant reduction in the population numbers of this species has occurred in sites that had previously been classed as having ‘good prognosis of recovery’ only 7 years ago. Whilst *A. palmata* was found in all the MPA zones surveyed, 30% of the total area currently occupied by *A. palmata* overlaps with those frequently exposed to localised threats such as lobster fishing and tourism. This suggests that there is an urgent need to reinforce and revise the management plans within the MPA to keeping away *A. palmata* populations at least from local threats.

## Supplemental Information

10.7717/peerj.1539/supp-1 Supplemental Information 1 *A. palmata* survey in Los Roques Croquer_etal.

## Additional Information and Declarations

### Field Study Permissions

The following information was supplied relating to field study approvals (i.e., approving body and any reference numbers): Ministerio del Poder Popular para el Ambiente, Oficina Nacional de Diversidad Biológica. Oficio No. 0323. Territorio Insular Francisco de Miranda. Jefatura de Gobierno. Permiso No. 006.

### Data Availability

The following information was supplied regarding data availability: The raw data was supplied in Supplemental Information 1.
